# Pharmacokinetics of single oral dose trazodone: a randomized, two-period, cross-over trial in healthy, adult, human volunteers under fed condition

**DOI:** 10.3389/fphar.2015.00224

**Published:** 2015-10-02

**Authors:** Prashant Kale, Yadvendra K. Agrawal

**Affiliations:** ^1^Lambda Therapeutic Research LimitedAhmedabad, India; ^2^Department of Research and Development, Gujarat Forensic Sciences UniversityGandhinagar, India

**Keywords:** bioequivalence, trazodone, pharmacokinetics, clinical trial, LC-MS/MS

## Abstract

**Objective:** To assess the bioequivalence of single dose trazodone hydrochloride USP 100 mg tablets administered as an oral dose under fed condition.

**Methods:**This study was an open-label, balanced, randomized, two-sequence, two-treatment, two-period, single oral dose, crossover bioequivalence study in healthy, adult, human subjects under fed conditions. After an overnight fast of at least 10 h, the subjects were served a high fat and high calorie vegetarian breakfast, which they were required to consume within 30 min. A single oral dose (100 mg) of either the test or the reference product was administered to the subjects. The primary pharmacokinetic parameters, maximum plasma concentration (C_max_) and area under the plasma concentration–time curve (AUC) from time zero to last measurable concentration (AUC_0−*t*_) and extrapolated to infinity (AUC_0−∞_) were compared by an analysis of variance using log-transformed data. Bioequivalence was concluded if the 90% confidence intervals (CIs) of the adjusted geometric mean (gMean) ratios for C_*max*_ and AUC were within the predetermined range of 80–125%, in accordance with regulatory requirements.

**Results:**For the test formulation, the trazodone gMean C_max_ was 1480.9 ng/mL (vs. 1520.2 ng/mL for reference), AUC_0−*t*_ was 18193.0 ng·h/mL (vs. 18209.8 ng·h/mL) and AUC_0−∞_ was 19346.3 ng·h/mL (vs. 19393.4 ng·h/mL). The 90% CIs for the ratio (test/reference) were 93.0–102.0% for C_max_, 96.7–103.2% for AUC_0−*t*_ and 96.1–103.5% for AUC_0−∞_. There were no deaths or serious adverse events during the conduct of the study.

**Conclusion:**Test product when compared with the Reference product meets the bioequivalence criteria with respect to the extent of absorption of trazodone under fed condition.

## Introduction

Trazodone Hydrochloride is an antidepressant chemically unrelated to tri-cyclic, tetra-cyclic, or other known antidepressant agents. The mechanism of trazodone hydrochloride's antidepressant action in man is not fully understood. In animals, trazodone selectively inhibits its serotonin uptake by brain synaptosomes and potentiates the behavioral changes induced by the serotonin precursor, 5-hydroxytryptophan. Trazodone is not a monoamine oxidase inhibitor and, unlike amphetamine-type drugs, does not stimulate the central nervous system. Trazodone is rapidly and almost completely absorbed from the GI tract following oral administration. The rate and extent of absorption are affected by the presence of food. Peak plasma concentrations of trazodone occur approximately 1 h after oral administration when the drug is taken on an empty stomach or 2 h after oral administration when taken with food.

When trazodone is taken shortly after the ingestion of food, there may be a slight increase (up to 20%) in the amount of drug absorbed. Distribution of trazodone into human body tissues and fluids has not been determined. Plasma concentrations of trazodone decline in a biphasic manner. Following oral administration of single doses of 25, 50, or 100 mg of trazodone to healthy, fasted adults in another study, mean peak plasma trazodone concentrations were 490, 860, and 1620 ng/mL, respectively (Nilsen and Dale, 1992). The half-life of trazodone in the initial phase is about 3–6 h and the half-life in the terminal phase is about 5–9 h. The clearance of trazodone from the body shows wide inter-individual variations. The therapeutic range for plasma trazodone concentrations and the relationship of plasma concentrations to clinical response and toxicity have not been established.

As per another reported(Gammans et al., 1984) study, following single doses, trazodone is rapidly and completely absorbed(Jauch et al., 1976; Ankier et al., 1981; Cassia et al., 1982) with peak plasma concentrations achieved 0.5–2 h following administration. The mean elimination half-life of trazodone has been variously reported as 4 h (Ankier et al., 1981) and 12 h (Jauch et al., 1976). Trazodone is eliminated primarily by metabolism(Biocchi et al., 1974).

The aim of the study was to compare the bioavailability and assess the pharmacokinetic profile of the test formulation Trazodone Hydrochloride tablets USP 100 mg manufactured by Intas Pharmaceuticals Ltd., India in comparison with the reference formulation TrazoDONE Hydrochloride tablets USP 100 mg manufactured by Apotex Inc., Toronto, Canada.

## Subjects and methods

This was a single-center (Lambda Therapeutic Research Ltd, Ahmedabad, India), randomized, single-dose, open-label, 2-treatment, 2-period, 2-sequence, crossover trial conducted in healthy volunteers between October and November 2013. The Independent Ethics Committee reviewed the study Protocol, Informed consent Form, Curriculum Vitae of Investigators and Product information (American Society of Health-System Pharmacists Inc, 2009). The study was conducted in accordance with local regulations and the Declaration of Helsinki and Good Clinical Practice (Guideline for Good Clinical Practice, 1996; Declaration of Helsinki, 2008). Written informed consent was obtained from all subjects before performing any trial-related activities. For this bioequivalence study, a crossover design was planned as per the USFDA requirements (US Department of Health and Human Services, Center for Drug and Research, 2003). A crossover study was conducted on 56 subjects under fed conditions.

### Subjects

All subjects willing to participate in the study were screened prior to their enrolment, in order to assess their eligibility by satisfying all of the inclusion and exclusion criteria. During screening, the medical history of the subjects was elicited and they underwent a general clinical examination, measurement of blood pressure, heart rate, oral body temperature, respiratory rate, 12-lead Electrocardiogram (ECG), clinical laboratory evaluations, chest X-ray and immunological tests for Human Immunodeficiency Virus (HIV), Hepatitis B Surface antigen (HBsAg), Hepatitis C Virus (HCV). This procedure was conducted within 21 days prior to the first dose of investigational medicinal product (IMP) administration. Healthy human subjects aged 18–45 years with a body mass index (BMI) of 18.5–27.5 calculated as kg/(height in m)^2^ were considered for the study. Additional exclusion criteria included any history or presence of asthma or nasal polyp or any other non-steroidal anti-inflammatory drugs (NSAID)—induced urticaria, smokers, who smoked >10 cigarettes per day or donation of blood (350 ml), or receipt of an IMP or participation in a drug research study within 90 days prior to receiving the first dose of study medicine. No female volunteers were enrolled in the study.

### Study design and treatments

In this study the screening phase was carried out within 21 days prior to the scheduled dosing day of Period-I. The duration of the clinical part of the study was about 8 days (11 h prior to the dose administration in Period-I until the last pharmacokinetic sample in Period-II). Based on available and data and statistical analysis and elimination half-life of trazodone, a wash out period of 4 days was kept in between the dosing days of the study periods. In this study with a crossover design, each subject received both the treatments (test drug and reference drug) during the study. Hence, every subject acted as his own control and no separate group of subjects was required to act as the control group. The sequence of administration was determined by the randomization schedule.

After an overnight fast of at least 10 h, the subjects were served a high fat (~50% of total caloric content of the meal) and high calorie (~800–1000 calories) vegetarian breakfast consist of 65 g of Carbohydrates (260 Kcal), 60 g of Fats (540 Kcal), and 34 g of Proteins (137 Kcal) resulting into total calories (Kcal) of 937, which they required to consume within 30 min. A single oral dose (100 mg) of either the test or the reference product was administered to the subjects at 30 min after serving the breakfast. The IMP was administered in sitting posture with 240 mL of drinking water at ambient temperature.

This activity was followed by a mouth check to assess the compliance to dosing. All the subjects were instructed to remain in sitting posture or ambulatory position for the first 3 h after administration of IMP in each period. During each period subjects remained in the study center until blood samples had been taken 48 h after dosing.

### Pharmacokinetic evaluation

Blood samples were collected through an indwelling intravenous cannula (Venflon) placed in a forearm vein of the subjects. A total of 23 blood samples, each of 05 mL were collected from each subject in each period at pre-dose (0.0 h) and at 20 min, 40 min and at 1, 1.25, 1.5, 1.75, 2, 2.25, 2.5, 2.75, 3, 3.5, 4, 5, 6, 8, 10, 12, 16, 24, 36, and 48 h following drug administration. Immediately after collection of blood, the collection tube (vacutainer) was inverted gently several times to ensure the mixing of tube contents [i.e., anticoagulant Dipotassium Ethylene Diamine Tetraacetic Acid (K2EDTA)]. The blood samples were centrifuged at 3000 ref for 5 min below 10°C to separate plasma. The separated plasma was stored in a freezer below −55°C.

The plasma samples of subjects were analyzed using a validated LC-MS/MS method (Kale et al., 2014) for trazodone at the Bioanalytical facility of Lambda Therapeutic Research Ltd., Ahmedabad, India. The method was validated as per the regulatory guideline (US Department of Health and Human Services, Food and Drug Administration, 2001). Calibration curves using an eight-point calibration curve standards for trazodone, with concentration ranging from 5.2 ng/mL to 3025.2 ng/mL, were used to determine the concentration of Trazodone in the samples of various subjects.

Plasma concentrations of trazodone were analyzed using liquid chromatography mass spectrometry procedures, which were fully validated and developed at Lambda Therapeutic Research Ltd, Ahmedabad, India. Briefly, trazodone plasma concentrations were measured following protein precipitation extraction (internal standard trazodone-d6) by liquid chromatography mass spectrometry (LC-MS/MS) using a Eclipse XBD C8 150 X 4.6 mm, 5 μm column, (mobile phase 70% methanol and 30% 2 mM Ammonium Format buffer pH 3.0). The limit of quantification was 5.2 ng/mL. Assay performance was assessed by back-calculation of calibration standards, tabulation of the standard curve fit function parameters and measurement of quality control (QC) samples. In all the cases, analytical run was rejected or accepted on the basis of the results obtained for QC samples run with that particular analytical run. There were four levels of QCs spread across the calibration curve range analyzed with each run in duplicates. The inter-day precision and accuracy of QCs during the study were from 2.0–7.7 to 100.2–102.9%, respectively, which were within the acceptance range of ±15% from the nominal value. Validation data documented adequate accuracy, precision and specificity of the liquid chromatography mass spectrometry assays employed for the study.

A total of 2169 samples were analyzed during the study. Out of these, 231 samples were reanalyzed to establish incurred sample reproducibility (ISR). Out of the incurred samples selected per subject, at least one sample was close to the maximum concentration and another sample was near the elimination phase to cover the entire concentration range. A total of 223 (96.5%) samples were within the acceptance criteria of ±20% differences between two values.

### Safety evaluation

All the subjects underwent a pre-enrolment laboratory parameters evaluation including tests for hematology, biochemistry, immunology, and urine analysis. The post-study safety assessments included hematology and biochemistry (except random glucose, sodium, potassium, and chloride). Sitting blood pressure and radial pulse were measured during each clinical examination, prior to administration of study drug and at approximately 02 and 11 h after administration of IMP in each period. Subjects were questioned for well-being at the time of clinical examinations and at the time of recording of vital signs in each period. Adverse events were collected during each study period with severity (mild, moderate, or severe) and investigator assessment of the relationship to the study medication (definite, possible, doubtful, or none).

### Pharmacokinetic analyses

The pharmacokinetic parameters were derived individually for each analyzed subject from the concentration vs. time profiles of trazodone in plasma. The primary variables were the area under the plasma concentration-time curve from time 0 to the last quantifiable data point (AUC_0−*t*_), from time zero extrapolated to infinity (AUC_0−∞_) and C_max_. Time of maximum exposure (*t*_max_) was a secondary variable. Non-compartmental analysis of plasma concentration-time data was performed using WinNonlin® Professional software (Version 5.3, Pharsight Corporation, USA). Actual time points of the sample collection were used for the calculation of pharmacokinetic parameters. All values below the limit of quantification were considered as zero for pharmacokinetic analysis.

### Statistical analyses

Descript statistics were computed and reported for primary and secondary pharmacokinetic parameters for trazodone. Analysis of variance was performed using PROC MIXED (SAS®, version 9.3, SAS Institute Inc., USA^1^) for ln-transformed pharmacokinetic parameters AUC_0−∞_, AUC_0−t_ and C_max_ for trazodone. The ANOVA model included sequence, period and formulation as fixed effects and subject (sequence) as a random effect. Using two one-sided tests for bioequivalence, 90% confidence intervals (CIs) for the ratio of geometric least squares means between drug formulations were calculated for ln-transformed pharmacokinetic parameters AUC_0−∞_, AUC_0−t_, and C_max_ for trazodone. Bioequivalence was concluded if the 90% CIs were within the range 80–125%. For all other parameters, descriptive statistics were presented. The power of the study to detect 20% difference between the test and reference formulations was computed and reported for trazodone.

## Results

A total of 58 subjects were enrolled and checked in for the study. As per the protocol 56 subjects were randomized [mean (standard deviation) 30.2 ± 6.1 years, BMI 22.1 ± 2.3 kg/m^2^] in period-I of the study. Two subjects were withdrawn from the study on medical grounds and two on the grounds of emesis in Period-I. Five subjects were withdrawn from the study on medical grounds in Period-II. In all, 47 subjects completed the clinical phase of the study successfully.

**Figure 1:**
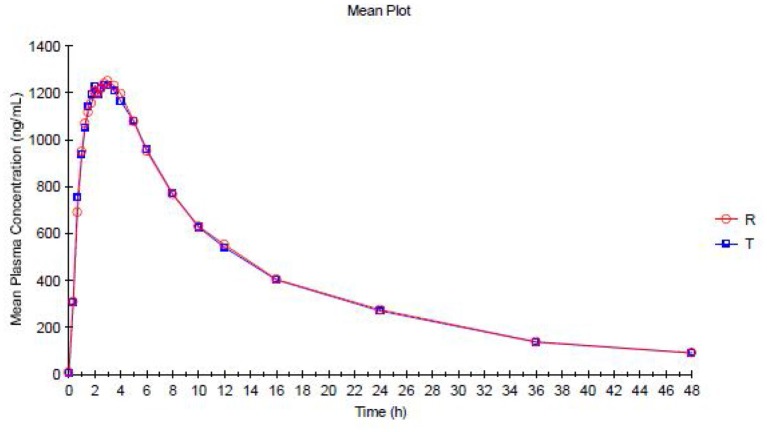
**Mean Plasma concentration vs. Time curve for Trazodone (Test Product—T and Reference Product—R)—Linear plot**.

**Figure 2:**
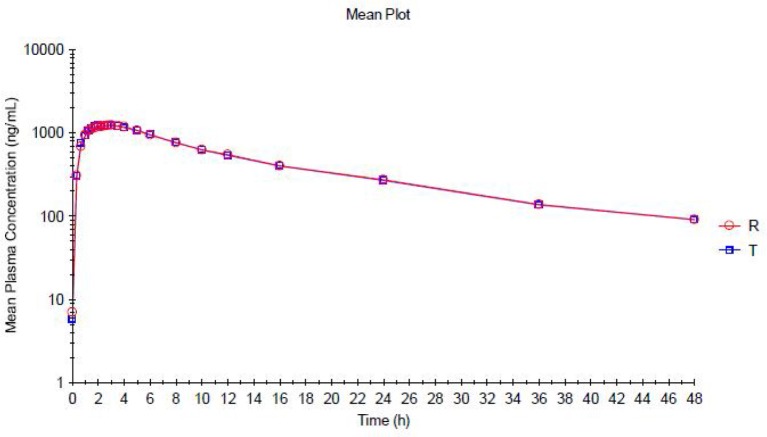
**Mean Plasma concentration vs. Time curve for Trazodone (Test Product—T and Reference Product—R)—Semi logarithmic Plot**.

The trazodone plasma concentration-time profiles are shown in Figures 1, 2 and pharmacokinetic parameters are summarized in Tables 1, 2.

**Table 1:** **Descriptive statistics of formulation means for Trazodone**.

**Parameters (units)**	**Mean ± SD (un-transformed data)**	
	**Test product-T**	**Reference product-R**
*T*_max_ (h)^*^	2.0 (0.667–6.02)	2.25 (0.667–6.0)
C_max_ (ng/mL)	1521.8 ± 364.5	1546.9 ± 315.4
AUC_0−*t*_ (ng.h/mL)	18864.6 ± 5374.1	18982.8 ± 5217.2
AUC_0−∞_ (ng.h /mL)	19959.8 ± 5442.8	20358.9 ± 5869.1
*t*_½_ (h)	12.9 ± 3.9	13.1 ± 3.5

* T_max_ is represented as median (min-max) value.

**Table 2:** **Relative bioavailability results for Trazodone**.

**Parameters**	**Geometric least squares means**			**90% confidence interval**
	**Test product-T**	**Reference product-R**	**Ratio (T/R)%**	
lnC_max_	1480.9	1520.2	97.4	93.01–102.04
lnAUC_0−*t*_	18193.1	18209.8	99.9	96.71–103.22
lnAUC_0−∞_	19346.3	19393.4	99.8	96.10–103.55

Trazodone was rapidly absorbed, with a median *t*_max_of 2 h (Table 1). The plasma concentration-time curves of trazodone showed a parallel decline in distribution and elimination phases (Figure 1). The gMean values of C_max_, AUC_0−*t*_ and AUC_0−∞_ for trazodone were comparable for test and reference formulations with low inter-individual variation (CV ranged from 9.2 to 13.1%). Bioequivalence was demonstrated as the 90% CIs of the ratios of point estimates (test/reference) for C_max_, AUC_0−*t*_ and AUC_0−∞_ were within the range of 80–125%.

### Safety results

In general, the clinical portion of the study was completed with nine (09) significant adverse events which resulted into withdrawal of the subjects from the study with the reasons Giddiness and vomiting; drowsiness; upper respiratory tract infection; chest pain; fever and headache; toothache and fever. The investigational products were well tolerated by healthy subjects, as a single dose administration. Twelve (12) adverse events (AEs) were reported by ten (10) subjects during the conduct of the study. Eleven (11) AEs were mild in nature and one (01) AE was moderate in nature. The subjects were treated accordingly and were followed up until resolution of their AEs. The causality assessment was judged as possible for four (04) AEs, as unlikely for three (03) AEs and as unrelated for two (02) AEs.

There were no deaths or serious adverse event during the conduct of the study. There were no clinically significant findings in the vital signs assessment or the laboratory tests in any of the subjects.

## Discussion

The ratio of geometric least squares means of Test product and Reference Product for ln-transformed pharmacokinetic parameter, Cmax was 97.4%. The 90% CIs for the ratio of geometric least squares means was found to be 93.01–102.04%. The ratio of geometric least squares means of Test product and Reference Product for ln-transformed pharmacokinetic parameter, AUC_0−*t*_ was 99.9%. The 90% CI for the ratio of geometric least squares means was found to be 96.71–103.22%. The ratio of geometric least squares means of Test product and Reference Product for ln-transformed pharmacokinetic parameter, AUC_0−*t*_ was 99.8%. The 90% CI for the ratio of geometric least squares means was found to be 96.10–103.55%. These intervals were within the acceptance limits of 80.00–125.00%, required for the conclusion of bioequivalence as per criteria set in the protocol.

Upon conclusion of the clinical portion of the study, the results from all subjects, who completed post-study procedures including laboratory tests and vital signs measurements, confirmed the absence of significant changes in the subject's state of health.

In summary, Test Product when compared with the Reference product meets the bioequivalence criteria with respect to the rate and extent of absorption of Trazodone under fed conditions as per criteria set in the protocol.

For the test formulation, the trazodone gMean C_max_ was 1480.9 ng/mL (vs. 1520.2 ng/mL for reference) which is comparable to the published data 1470 ng/mL (Nilsen and Dale, 1992) where as in another study it was 1188 ng/mL after 300 mg extended release tablet (Jessica and Alan Caspi, 2011). In this study AUC_0−*t*_ was 18193.0 ng·h/mL (vs. 18209.8 ng·h/mL) and AUC_0−∞_ was 19346.3 ng·h/mL (vs. 19393.4 ng·h/mL).

In another study the same drug was tested under fasting conditions. For the test formulation, the trazodone gMean C_max_ was 2172.2 ng/mL (vs. 2031.2 ng/mL for reference), AUC_0−*t*_ was 16631.6 ng·h/mL (vs. 16342.9 ng·h/mL) and AUC_0−∞_ was 17460.6 ng·h/mL (vs. 17270.1 ng·h/mL) (Kale and Agrawal, 2015).

### Conflict of interest statement

The reviewer, “Martin Michel” declares that, despite having collaborated with the author “Prashant Kale,” the review process was handled objectively and no conflict of interest exists. This study was sponsored by Intas Pharmaceuticals Limited, India. The author is an employee of Lambda Therapeutic Research Limited which was contracted by Intas as CRO for conduct of this study. The authors have no financial involvement or financial conflict with the subject matter or materials discussed in the manuscript.

## Footnotes

^1^SAS/STATR User's Guide. SAS Institute Inc., USA, Version 9.3.
